# New subgenus and new species of Oriental *Omophorus* (Coleoptera, Curculionidae, Molytinae, Metatygini)

**DOI:** 10.3897/zookeys.85.973

**Published:** 2011-03-07

**Authors:** Zhiliang Wang, Miguel A. Alonso-Zarazaga, Li Ren, Runzhi Zhang

**Affiliations:** 1 Key Laboratory of Zoological Systematics and Evolution, Institute of Zoology, Chinese Academy of Sciences, No. 1 Beichen West Road, Chaoyang District, Beijing 100101, China; 2 Departamento de Biodiversidad y Biología Evolutiva, Museo Nacional de Ciencias Naturales (*CSIC*); 3 Graduate University of Chinese Academy of Sciences, 19A Yuquan Road, Shijingshan District, Beijing 100049, PR China

**Keywords:** Weevils, morphology, systematics, phylogeny, *Sinomophorus*, new species, Yunnan, China

## Abstract

The genus Omophorus Schoenherr, 1835 is recorded for the first time from the Oriental Region and a new subgenus and species, Omophorus (Sinomophorus subgen. n.) rongshu **sp. n.** is described from Yunnan province (P.R. China). The new subgenus differs from the subgenus Omophorus by the longer antennal club, the bifid vestiture of the ventral parts, the elongate subtrapezoidal scutellum, the very small size of sclerotizations in the endophallus, the absence of styli in the ovipositor and the absence of the spiculum ventrale on the VIII female sternite, and from the subgenus Pangomophorus Voss, 1960 by the developed metatibial uncus and the lack of a subhumeral tubercle. A detailed description and figures are provided to allow interpretation of characters in ongoing phylogenetic analyses.

## Introduction

The superfamily Curculionoidea Latreille, 1802 is a very speciose group of Coleoptera, and probably the family Curculionidae Latreille, 1802 (in its different limits) the most speciose in the Animal Kingdom; weevils, as they are known, currently comprise some 62,000 known species and an approximate analysis ranks the number of total species close to 220,000 (Oberprieler et al. 2007). Consequently it is not strange to find undescribed taxa, particularly in provinces like Yunnan (P.R. China), a territory including part of two biodiversity hotspots, the Indo-Burman and the Mountains of Southwest China (Mittermeier et al. 2000).

The tribe Metatygini was named by Pascoe (1888) as a subfamily of Curculionidae and included the type genus Metatyges Pascoe, 1865 (now a junior synonym of Omophorus Schoenherr, 1835) and the genus Zantes Pascoe, described as new then, apparently on the grounds only of the sharing of the “short outline” of the body in dorsal view.

Alonso-Zarazaga and Lyal (1999) placed the tribe Metatygini Pascoe, 1888 in the subfamily Molytinae Schoenherr, 1823 and recognised five genera in it, namely, Omophorus Schoenherr, 1835, Physarchus Pascoe, 1865, Sternechosomus Voss, 1958, Teluropus Marshall 1917 and Zantes Pascoe, 1888. The last two are not members of this tribe and should be placed elsewhere (C.H.C. Lyal and R. Oberprieler, pers. comm.). Physarchus and Sternechosomus differ from Omophorus by the presence of projecting dentiform humeral calli, and also by the presence of 2 apical teeth on the underside of onychia (C.H.C. Lyal and R. Oberprieler, pers. comm.). At present, the genus Omophorus includes 5 African species in the subgenus Omophorus s. str. and one Papua New Guinean species in the subgenus Pangomophorus Voss, 1960. Physarchus includes 3 Oriental and Polynesian species,, and Sternechosomus includes one species from Fujian (China) (see Appendix I for a species checklist with distributional data). However, some other undescribed species in the three genera have been found in collections (H. Kojima, C.H.C. Lyal, R. Oberprieler, pers. comm. and unpublished data). Even if Teluropus is not considered contribal, an evaluation of its placement should take into consideration that its type species has been recorded as damaging buds of Artocarpus heterophyllus (Moraceae) (Jha and Sen-Sarma 2008), the same family of plants on which Omophorus feeds (Marshall 1944; Taylor 1978).

The discovery of a new member of this tribe in the collections of the Institute of Zoology of the Chinese Academy of Sciences (IOZ-CAS), that we consider a new species of Omophorus, and a second representative of this tribe in China, leads us to the following description.

## Materials and methods

Specimens of a new species were found while sorting out and identifying specimens in the collection of IOZ-CAS (Beijing, P.R. China). Some of them were dissected after soaking them overnight in lukewarm soapy water and later rinsed with de-ionized water. Lukewarm 10% potash solution overnight was used for digestion of soft tissues. One defective male was completely dissected and mounted to study and photograph notal areas, metendosternite, wings, terminalia and genitalia. Description of these parts follows Davis (2009) and the references therein. Metendosternite nomenclature follows Velázquez de Castro (1998) and that of wings follows Zherikhin and Gratshev (1995); other special terminology follows the “Glossary of characters” hosted on the International Weevil Community Website (http://weevil.info).

The holotype of Omophorus rongshu has not been dissected to avoid damage, since there were enough specimens collected with it. The holotype and some paratypes have been unpinned and glued to bristol pointcards, their parts mounted in DMHF (5,5-dimethyl hydantoin formaldehyde resin) in acetate cards pinned under each specimen. Other paratypes are as they were mounted after collecting, pinned through the right elytron base.

Descriptions were made using a binocular Zeiss Stemi SV11. Photographs were taken with a CCD Qimagine MircoPublisher 5.0 RTV mounted on a Zeiss SteREO Discovery V.12. Extended focus images were generated with Auto-Montage Pro 5.03.0061 and edited with Adobe Photoshop CS 5.0 if required. Microscopic slides were studied under a Leica DM 2500 microscope and photos were taken with a Nikon CoolPix 5400.

Label data are given as they are (in Chinese), with pinyin romanization and comments in square brackets; labels are separated by semicolons and lines by slashes.

## Taxonomic treatment

### Genus Omophorus

Schoenherr, 1835

#### Type species.

Omophorus stomachosus Boheman, 1835. For a complete synonymy, check Appendix I. This genus includes two subgenera: Omophorus s.str., with five species showing an Afrotropical distribution and Pangomophorus Voss, 1960, with a single species described from Papua New Guinea. A new subgenus and species from China are described below:

#### Sinomophorus subgen. n.

urn:lsid:zoobank.org:act:D1F53C57-971E-40DF-9105-DE5096B54ADC

##### Type species.

Omophorus rongshu sp. n.

##### Diagnosis.

It differs from subgenus Omophorus s.str. by the longer antennal club, the bifid vestiture of the ventral parts, the elongate subtrapezoidal scutellum, the very small size of sclerotizations in the endophallus, the absence of styli in the ovipositor and the absence of a spiculum ventrale on the VIII female sternite, and from subgenus Pangomophorus by the metatibial uncus developed and the lack of a subhumeral tubercle.

##### Etymology.

From Latin *Sina* (China) and genus Omophorus. Gender masculine, as Omophorus.

##### Omophorus (Sinomophorus) rongshu sp. n.

urn:lsid:zoobank.org:act:9173392E-0DAE-421C-A8CF-EF0E39C1C871

**Figures 1–2.**
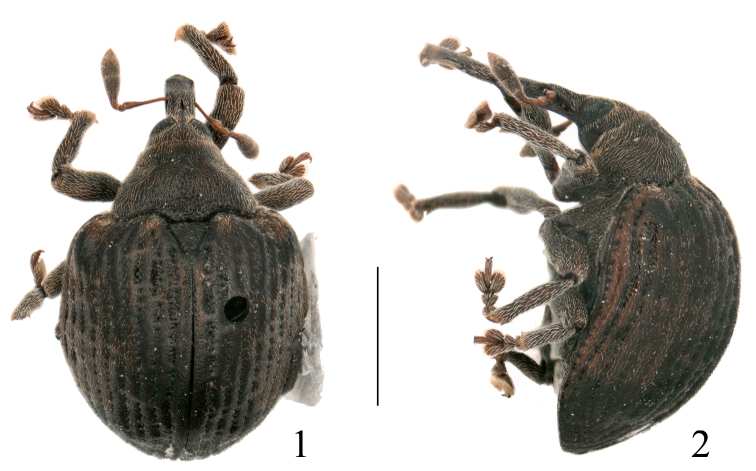
Omophorus (Sinomophorus) *rongshu* sp. n., male holotype, habitus. **1** dorsal view **2** lateral view. Scale: 1–2: 2 mm.

Figs 1 [Fig F2] [Fig F3] [Fig F4] [Fig F5] [Fig F6] 23

###### Description

(holotype, except where indicated).

####### Measurements (in mm):

Standard length: 4.95. Rostrum: length: 1.08, maximum width: 0.43. Pronotum: median length: 1.09, maximum width: 2.01. Elytra: median length: 4.10, maximum width: 3.70.

####### Integument.

Reddish brown, base and apex of elytra, a broad sutural band, venter of femora, parts of meso- and metasternum and their pleurites, and part of the 1^st^ ventrite dark brown; apical margin of 2^nd^-4^th^ ventrites and the whole 5^th^, antennal scape and funicle testaceous.

####### Vestiture

on elytra of scarce, short, very fine, pale piliform scales, most numerous on extreme base of 2^nd^-3^tr^ interstriae, on head and pronotum formed by the same scales, but denser and thicker, creamy to tan, on disc directed cephalad, on sides directed to midpoint of pronotum; on legs as on head and rostrum, but larger and whiter; on ventral areas, coxae, trochanters and ventral part of femora, vestiture of bifid piliform scales, each scale with a common stalk hardly longer than basal width of each branch. Sclerolepidia absent. Remnants of waxy-pulverulent, creamy to tan exudate in some parts of elytra and pronotal base.

####### Rostrum

**Figures 3–8. F2:**
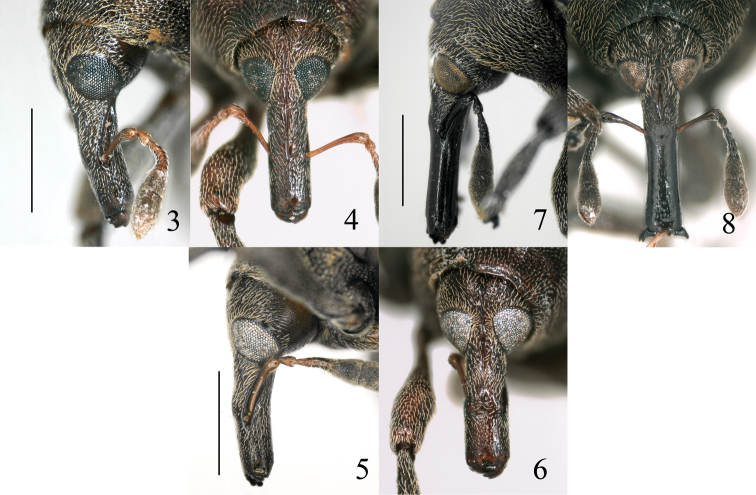
Omophorus (Sinomophorus) *rongshu* sp. n., head and rostrum. **3** male holotype, dorsal view **4** male holotype, lateral view **5** male from Xīnpíng, dorsal view **6** male from Xīnpíng, lateral view **7** female paratype, dorsal view **8** female paratype, lateral view. Scale: 3–8: 1 mm.

(Figs 3–4) 0.99 × as long as pronotum, in dorsal view 2.51 × as long as wide, with apex slightly bisinuate, sides subparallel, densely punctate, except smooth and shiny apical tenth, metarostrum with a median sulcus prolongated basad to hind margin of eye, ending apically at antennal insertion level; in side view metarostrum dorsally depressed, prorostrum a little narrowed to apex, ventral margin straight, forming a soft curve with underhead. Scrobes dorsally visible at apex, in side view almost straight, deep, narrow, upper margin directed towards lower angle of eye, but not reaching it, becoming obsolete, lower margin convergent with upper and evanescent; scrobes in ventral view not convergent, obsolescent against under margin of eye. Underside of rostrum densely punctate, with three low keels, the lateral ones ending at insertion level, the median one distally fusing with the submentum. Labium ca. 1.5 × as long as wide, sides weakly rounded, medially longitudinally impressed, apically truncate to weakly emarginate, 1.5 × as long as labial peduncle, labial palps invisible; maxillary palp 3-segmented. Mandibles tridentate, overlapping. No postmandibular sensory setae.

####### Head

in dorsal view subglobular, densely and subrugosely punctate, medially sulcate up to occiput, frons narrow, ca. 0.5 × as wide as rostral apex, in side view frons weakly convex; eyes moderately large, slightly convex, ca. 1.5 × higher than long, transversely elliptical, some 16 ommatidia in longitudinal diameter.

####### Antennae

inserted at basal 0.49 of rostrum, with scape 5.58 × as long as wide and 1.09 × as long as funicle, slightly clubbed and bent at apex, glabrous except pubescent apical club, 1^st^ desmomere 1.7 × as long as wide, obconical, well separated from 2^nd^, this also obconical and 1.56 × as long as wide, but slightly narrower than 1^st^ and 0.82 × as long as it, desmomeres 3^tr^–6^th^ 2.0 × as wide as long, tightly packed and increasing in width apicad, 7^th^ annexed to club, 3.0 × as wide as long and covered with a denser vestiture than others; club of 3 segments, suture between 1^st^ and second obliterated, almost invisible, that between 2^nd^ and 3^tr^ narrow but visible, so that apparently the club is bisegmented, last segment 1.3 × as long as remainder of club, whole club 2.54 × as long as wide, 1.69 × as long as funicle.

####### Pronotum

in dorsal view 1.85 × as wide as long, its base 1.79 × as wide as apex, bisinuate, with marginal keel, median lobe weakly and widely emarginate, surface densely punctate at base and on disc, punctures oblong, ca. 60 μm long, separated half their diameter or less, on sides and apical third smaller, 30 μm or less, widely spaced, a small median smooth and shiny tubercle just behind the faint collar constriction. Basal angles rounded-subtruncate.

####### Mesonotum

(Fig 18). Scutellum 0.82 × as long as wide, large, trapezoidal, wider at base, sides sinuate, apex subtruncate, slightly bituberculate, each tubercle with short setae, surface densely and rugosely punctate, punctures as small as those on apex of pronotum. Prephragma well developed, strongly prominent, antecostal sutures straight, meeting at middle in an obtuse V, mesoscutum smaller than scutellum. Axillary cord laterally prominent as a round lobe, joining in a soft curve the postero-lateral margin of mesonotum.

####### Metanotum

(Figs 14–15). In general shape, anterior part forming a vertical wall with a very narrow, transversal opening. Anterior margin of prescutum with a pair of prominent teeth and a deep median notch. Antero-medial margin of allocrista rounded. Scutellar groove wide, its anterior margin widely open with a small elevation on each side, without anterior bridge, with a median longitudinal obtuse elevation, not keeled. Metascutum about as large as metascutellum, separated from it by a straight postero-medial margin. Postero-lateral margin of metanotum with a strong, laterally and slightly caudally pointing tooth, and a round lobe in front of it bearing two teeth. Postnotum small, strongly transverse, well separated from metascutum and metascutellum.

####### Elytra

(Fig 1) 1.11 × as long as wide, base of interstriae 2–4 convergent and prominent cephalad, covering base of pronotum, humeri moderately convex, sides of elytra widening from under the calli, widest at middle, uniformly rounded to apex, apices very narrowly rounded separately, sutural angle small, subacute. Striae 10, widely sulcate, with rows of small inner punctures, forming irregular aggregates separated by bridges uniting interstriae, giving a moderately foveolate aspect, each puncture with a subarcuate seta hardly as long as half the interstrial ones, and finer; interstriae weakly convex, subequal in width, with 2–3 irregular rows of extremely minute punctures (less than 6 μm in diameter), surface almost smooth, weakly microreticulate, first interstria strongly microreticulate, transversally rugose, more convex behind scutellum. Striae at apex join 1+10, 2+9, 3+8, 4+5, 6+7, 10^th^ marginal and obsolete in apical fourth, with a strong outer keel in basal ¾, 11th interstria completely facing ventrally from base up to level of abdominal suture III; at base, 1^st^ shortened and reaching the level of apical third of scutellum, 2^nd^-4^th^ convergent towards basal lobe of elytron. Internal submarginal fold well developed, strongly curved. Sutural flanges dissimilar, that on the left elytron wider, in basal part turned vertical, that on the right elytron narrower, parallel to elytral surface in its whole length. Underside with small, inconspicuous superficial file of stridulatory system type 1 (Lyal and King 1996) near apex.

####### Metathoracic wing

**Figure 9–11. F3:**
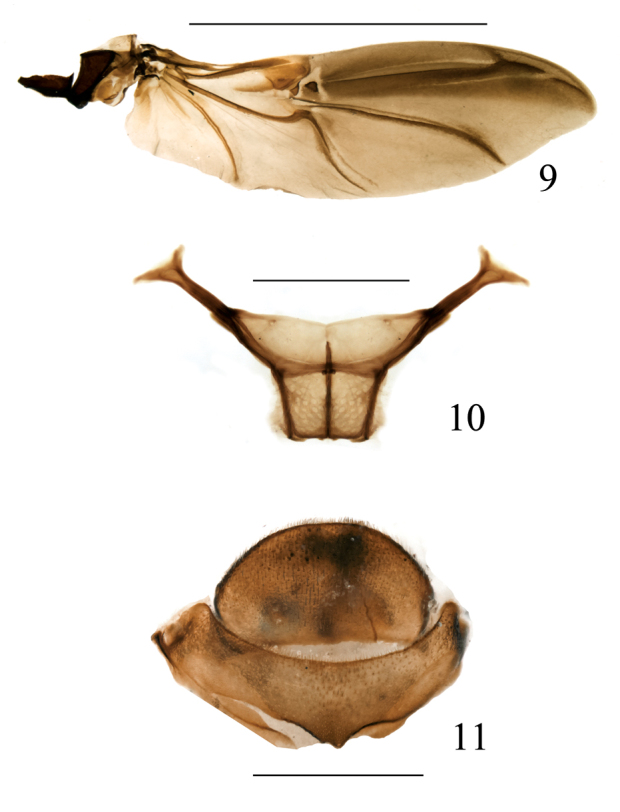
Omophorus (Sinomophorus) *rongshu* sp. n. **9** paratype, metathoracic wing **10** male paratype, metendosternite **11** female paratype, VII-VIII tergite, dorsal view. Scales: 9: 5 mm; 10–11: 1 mm.

(based on 2 paratypes) (Fig. 9). A typical curculionid wing, ca. 3.1 × as long as wide, with the surface covered with microtrichia. C, Sc and R without special characters. Rr undifferentiated in the apical half, rm and rms appearing as a single unit, rfi, rcm and rc forming a single plate in which rcm is weakly distinguishable as a small elongate trait. 1rs and 2rs form separate plates, united posteriorly to rsc. R3 present, thin, incomplete; anterior stripe thinner than the postradial stripe. M1 thin, parallel to mst, and joining it near apical third of mst length. Cu uniting to Cu1 with a strongly arched loop, Cu1 following to near margin as af. 1A1 and 1A2 absent. A strong, weakly arched, not reaching margin, 3A very weak, stripe-like, not closing ac. Basal sclerites: Br and Bsc fused.

####### Ventral area.

**Figures 12–18. F4:**
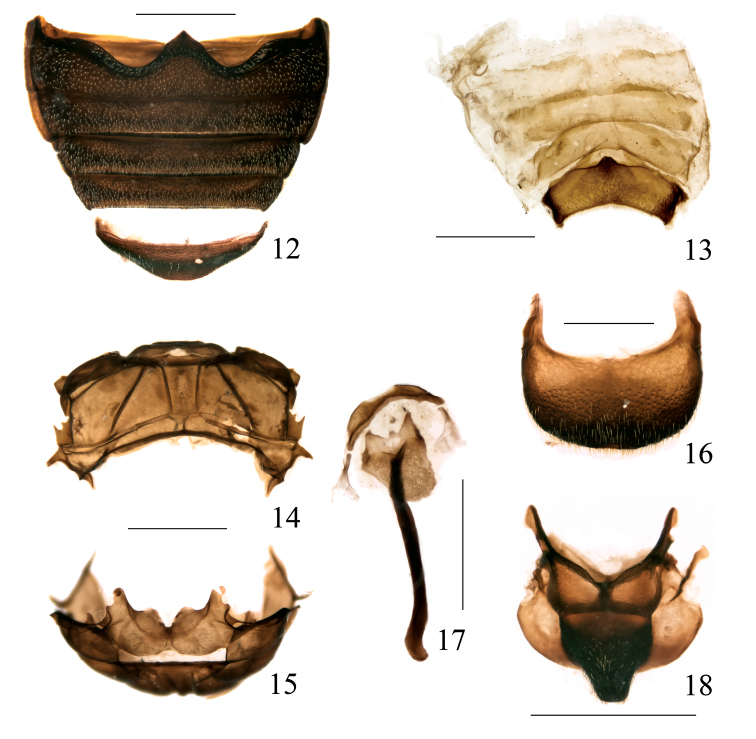
Omophorus (Sinomophorus) *rongshu* sp. n., male paratype. **12** ventrites, ventral view **13** tergites II-VII, dorsal view **14** metanotum, dorsal view **15** metanotum, frontal view (dorsum below) **16** tergite VIII, dorsal view **17** sternites VIII and IX, dorsal view **18** mesonotum, dorsal view. Scales: 12–15, 17: 1 mm; 16, 18: 0.5 mm.

Procoxal cavities separated from front margin of prothorax a distance twice that separating them from hind margin. Mesosternum very short, vertical in front, forming a steep angle in side view; meso-metasternal median suture not evident, replaced with a weak transverse sulcus; mesepisternum fused to mesosternum, mesosternal suture indicated by a small depression; mesepimeron triangular, fused to mesepisternum, mesopleural suture visible, anteriorly deepened, not functional. Metasternum fused medially to mesosternum, suture not visible, replaced with a weak sulcus, this joined in middle by a median longitudinal sulcus running from base to apex of metasternum, deeper at apex; a prominent transverse tubercle in front of metacoxae, forming a vertical step; metepisternum elongate-trapezoidal, apical angles a little produced laterally, the upper angle fitting in a small notch on the elytral costal margin, basal margin rounded; metasternal suture visible, complete, functional; metepisternum much reduced, covered by elytron. Abdomen (Fig 12) with 2^nd^ ventrite as long as 3^tr^ medially, 4^th^ shorter (0.9 ×), 5^th^ 1.19 × as long than 4^th^ and 4.97 × as wide as long, 1^st^ ventrite 1.94 × as long as second in median length, 1.11 × as long as 2^nd^ in postcoxal length. First ventrite strongly raised across basal margin, 5^th^ weakly depressed across middle. Suture I deep, weakly bisinuate, sutures II-IV functional, straight, slightly curved backwards at outer angles.

####### Metendosternite

(based on 1 paratype) (Fig 10): Stalk weakly trapezoidal and transverse, anterior part of longitudinal flange shorter than posterior, hemiductus robust, projection as long as wide, furcal arms long, apically clearly bifurcate, anteroventral branch shorter than laterodorsal one. Insertions of anterior tendons at the same distance as angles formed by margins of stalk and sheath.

####### Legs.

Procoxae contiguous, subconical. Mesocoxae separated by a distance 1.4 × the mesocoxal diameter. Mesocoxae and metacoxae separated 0.8 × the mesocoxal diameter. Metacoxae separated 0.5 × the distance between mesocoxae. Metacoxae transversely elongate, reaching the outer front angles of 1^st^ ventrite, outer part covered by meeting lobes of metasternum and 1^st^ ventrite, thus apparently very narrow. Trochanteral seta present. Femora edentate, robust (profemur 3.38 × as long as wide). Tibiae robust (protibia 4.06 × as long as wide), sides subparallel, inner margin very weakly bisinuate, not crenulate. Tibial unci raising from middle part of talus, leaving an outer angle and an inner praemucro, metatibial uncus shorter than others. Tibial comb complete, on almost transverse margin of talus. Front tibiae with a patch of dense golden setae near front inner apical angle, above comb, a grooming area also present in apical inner margin. Tarsomeres moderately robust, 1^st^ protarsomere 1.67 × as long as wide, 2^nd^ 0.73 ×, 3^tr^ 0.65 ×, with complete ventral sole, except for narrow glabrous midline, onychium 3.7 ×, surpassing lobes of 3^tr^ by 0.38 × length of onychium. Claws free, simple, divaricate, short and robust, as long as onychium height in side view.

####### Abdominal tergites

(based on 1 paratype) (Fig 13). Tergites I-III weakly sclerotized, submembranous; spiracular sclerites a little more sclerotized, round; tergites IV-VI more sclerotized, mostly in two marginal fasciae on each side of front margin; tergite VII (prepygidium) trapezoidal-semilunate, front median area with a transverse keel, the keel triangularly prominent at middle and apex of prominence bearing 2 small tubercles (plectra of stridulatory system type 1 of Lyal & King (1996)), the left one more developed than the right one, one transverse binding patch near the front angles; tergite VIII (pygidium) (Fig 16) more or less semicircular, front angles prominent, with one basal binding patch each, the apical area densely punctate, hispid.

####### Male genitalia and terminalia

**Figures 19–21. F5:**
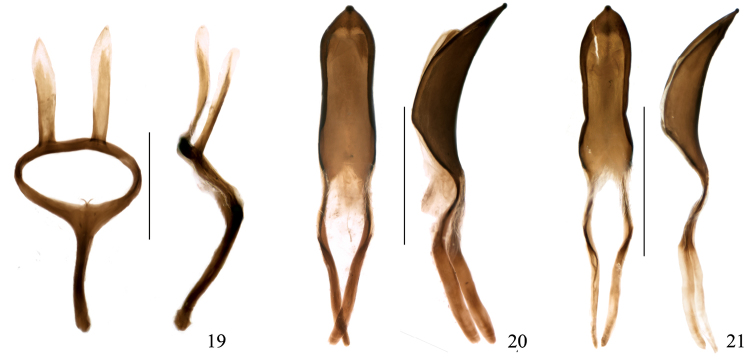
Omophorus (Sinomophorus) *rongshu* n.sp , male paratype. **19** tegmen, dorsal and lateral views **20** penis, dorsal and lateral views **21** male from Xīnpíng, penis, dorsal and lateral views. Scales: 19: 0.5 mm; 20–21: 1 mm.

(based on 1 paratype). Sternite VIII (Fig 17) undivided, semilunar, apex widely emarginate, more sclerotized basally, the membrane between this and the sternite IX with 2 very small, dot-like sclerotizations. Sternite IX with hemisternites fused between them and to the front margin of the spiculum gastrale to form an irregular subpentagonal plate, widely notched caudad; spiculum robust, directed towards left side of abdomen, almost straight, apex weakly hooked. Penis (Fig. 20) in dorsal view with tube 1.32 mm in length and temones 1.17 mm, tube ca. 3.2 × as long as wide, apex ogival, subacute, sides rounded, with a small apical peg, widest at basal 4^th^, temones fused; in lateral view moderately and uniformly curved at sides, highest at basal 3^tr^, apical peg rounded; a subtobtuse ventral longitudinal keel present. Endophallus without large sclerotized structures, covered with very dense, minute (2.5 μm long) teeth, some longer teeth forming several diffuse longitudinal streams. Tegmen (Fig. 19) with ring narrow, manubrium about as long as parameroid lobes, these widely spaced at base, lanceolate, slightly asymmetrical, apex and inner margin desclerotized, covered with minute translucid microchaetae, those at apex ca. 25 μm long.

###### Variation. Male paratypes.

Measurements (in mm) (n=7): Standard length: 4.40–5.30 (mean= 4.85). Rostrum: length: 1.08–1.22 (mean= 1.15), maximum width: 0.38–0.46 (mean= 0.42). Pronotum: median length: 0.91–1.20 (mean= 1.06), maximum width: 1.90–2.20 (mean= 2.05). Elytra: median length: 3.70–4.30 (mean= 4.00), maximum width: 3.05–3.95 (mean= 3.50).

*Rostrum* 0.98–1.11 × as long as pronotum, in dorsal view 2.57–2.88 × as long as wide, sides varying from subparallel to visibly but weakly widened towards apex in an almost straight line. *Pronotum* 1.79–2.09 × as wide as long in dorsal view. Scutellum with sides more or less straight and apex more or less rounder to weakly notched, the apical tubercles more or less separated and variably prominent. *Elytra* 1.08–1.25 × as long as wide.

###### Female paratypes.

Measurements (in mm) (n=7): Standard length: 4.60–5.30 (mean= 4.95). Rostrum: length: 1.42–1.56 (mean= 1.49), maximum width: 0.38–0.44 (mean= 0.41). Pronotum: median length: 1.04–1.28 (mean= 1.16), maximum width: 1.95–2.30 (mean= 2.13). Elytra: median length: 3.70–4.55 (mean= 4.13), maximum width: 3.20–3.90 (mean= 3.55).

As males, but *rostrum* (Figs 7–8) 1.21–1.37 × as long as pronotum, in dorsal view 3.55–3.74 × as long as wide, in dorsal view prorostrum glabrous, with sides gently curved, constricted at middle, minutely and sparsely punctate, punctures weak, metarostrum with sides weakly converging to mesorostrum, punctate and pubescent as in male; in side view straight, dorsal and ventral margins of prorostrum parallel; underside almost impunctate, subglabrous. *Head* with frons ca. 0.63 × as wide as rostral apex. *Antennae* inserted at basal 0.42 of rostrum, shorter, scape about as long as funicle, club 1.25 × as long as funicle.

*Pronotum* 1.79–1.95 × as wide as long in dorsal view. *Elytra* 1.05–1.25 × as long as wide.

*Tergites* VII and VIII (Fig 11) similar to those of male, tergite VIII less convex, semicircular, rather flat, with one small (functional?) spiracle on each side.

**Figure 22. F6:**
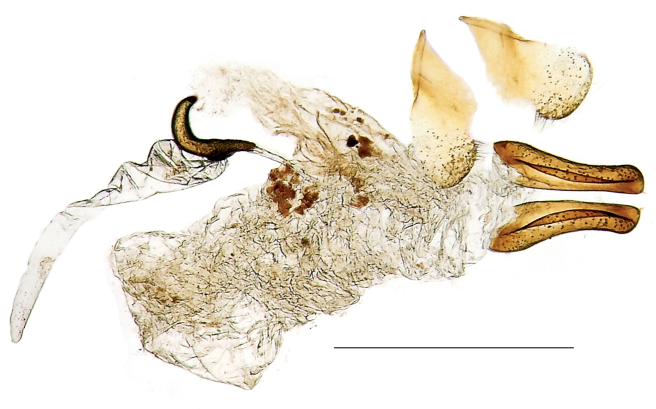
Omophorus (Sinomophorus) *rongshu* sp. n., female genitalia. Scale: 1 mm.

*Female genitalia and terminalia* (based on 2 paratypes) (Fig 22): Sternite VIII heart-shaped, with a wide apical sinus, most of it membranous and thin, laterally sclerotized, setose and with multiple sensilla, medially broadly membranose to base; basal convergent margins a little folded and sclerotized; spiculum absent. Ovipositor with gonocoxites robust (ca. 3.5 × as long as maximum width), moderately curved, weakly constricted at middle, rounded at apex, without styli, a small apicomedial fovea carrying a short, thick tuft of setae; dorsal surface with a membranous elongate triangular area, pointing apicad; ventral surface with a rounded longitudinal finlike flap; outer surface covered with moderately dense sensilla. Bursa irregular, large, wrinkled, with some small irregular sclerotized areas near the point of union of the spermathecal duct and of the oviduct. Spermatheca C-shaped, large (ca. 0.43 × as long as the 8^th^ sternum wide), with a long, apically rounded cornu not clearly differentiated from the spermathecal body (nodulus), except for the dorsal gibbosity, collum about as long as nodulus, ramus absent. Spermathecal gland ca. 3.2 × as long as maximum length of spermatheca.

###### Common variability.

The specimens show some variation in colour, mostly a lesser presence of the dark brown colouration on elytra, restricted to some basal spots and an apical patch, or to a lining of the bottom of the elytral striae, the apical margin of the ventrites, the sides of pronotum and the basal two thirds of the rostrum, the other areas remaining reddish brown, or all the head and rostrum may become also of the same colour. Legs may also show different variations. No variation has been noticed in vestiture, but the pronotal median tubercle may be more or less covered with punctures in different individuals.

###### Material examined.

Holotype: ♂: (white, handwritten): 云南路西 [Yúnnán Lùxī] / 1958.6.26; (white, handwritten): 中国科学院，榕树 [Zhōngguó Kēxuéyuàn, róngshù] 575; (white, printed): IOZ (E) 909962. Paratypes (12♂, 7♀): same data as holotype: 2♂ labeled IOZ(E) 909960, IOZ(E) 909961 and 1♀ labeled IOZ(E); 1♂, same data as holotype except date 1958.6.27 and IOZ(E) 909962 with same data as holotype except for collecting date: 1958.VI.27; 8♂, same data as holotype except date 1958.6.30 and IOZ(E) 909948, IOZ(E) 909949, IOZ(E) 909950, IOZ(E) 909954, IOZ(E) 909957, IOZ(E) 909958, IOZ(E) 909959, IOZ(E) 909963; 6♀, same data as holotype except date 1958.6.30 and IOZ(E) 909946, IOZ(E) 909951, IOZ(E) 909955, IOZ(E) 909956, labeled IOZ(E)1799147, IOZ(E)1799150; 1♂: (white, handwritten): 云南瑞丽 [Yúnnán Ruìlì] 1958.7.6; (white, handwritten): 中国科学院，榕树 [Zhōngguó Kēxuéyuàn, róngshù] 688; (white, printed): IOZ(E) 909964.

Holotype and all paratypes to be conserved in IOZ-CAS, except one male and one female paratype to be deposited in MNCN-CSIC (Madrid) and another paratype couple in the NHM (London).

The first character used to write the second place name (Lùxī) is incorrect. It should have been 潞.The only Yunnanese locality named 路西 is found at more than 2,000 m height.

###### Etymology.

The specific epithet is a pinyin transliteration (without diacritics) of the indication of the host plant on the labels, róngshù (榕树), which is applied to Ficus microcarpa L.f. (Moraceae) (Zhou and Gilbert, 2003). It is used as a noun in apposition.

###### Distribution and habitat.

**Figure 23.**
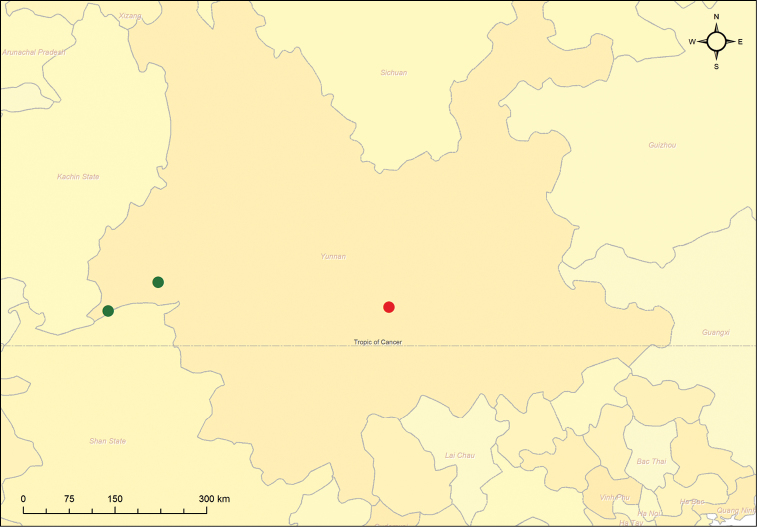
Omophorus (Sinomophorus) *rongshu* sp. n., distribution map. Red dot is Xīnpíng.

The species is known from two close localities in Yunnan province (P.R. China): Lùxī (575 m) and Ruìlì (688 m), separated by 87 km, but see below (Fig 23). No exact data about the biology of this species are known, except the identity of the host plant on which the specimens were captured, a common tree below 1900 m in the area, and widely planted as a shade tree (Zhou & Gilbert 2003).

###### Note.

One male has not been included in the type series, because it differs from the specimens collected in the two above mentioned localities as follows: *Rostrum* (Figs 5–6) more visibly widened towards apex, more robust (2.49 × as long as wide, 0.93 × as long as pronotum). Scape more robust (5.30 × as long as wide) but longer (1.23 × as long as funicle), 2^nd^ desmomere more robust (1.36 × as long as wide), 3^tr^-6^th^ desmomeres 2.7–3.7 × as wide as long, club more robust (2.39 × as long as wide) and shorter (1.42 × as long as funicle). *Pronotum* narrower, in dorsal view 1.67 × as wide as long. *Scutellum* as long as wide. *Abdomen* with 2^nd^ ventrite 1.2 × as long as 3^tr^, 4^th^ as long as 2^nd^, 5^th^ 4.65 × as wide as long, 1^st^ ventrite 1.66 × as long as 2^nd^ in median length, 0.89 × as long as 2^nd^ in postcoxal length. *Mesocoxae* separated by a distance 1.2 × the mesocoxal diameter. *Profemur* more robust, 3.0 × as long as wide, protibia more slender (5.43 × as long as wide. *Onychium* surpassing lobes of 3^tr^ tarsomere by 0.5 × length of onychium. *Penis* (Fig 21) similar, but more constricted medially in dorsal view, and ventral angle sharper (in cross-section).

Label data for this specimen are as follows: (white, two marginal faded red lines, pencil handwritten): 黑尾棕象甲 [Hēi Wěi Zōng Xiàng Jiǎ] / 新平 [Xīnpíng]; (white, faded red lined, field names printed, handwritten): 新平 [Xīnpíng];，80年; (white, printed): IOZ(E)1799122. The first label corresponds to an intent of identification in Chinese (the brown weevil with black tail) and the locality; the second repeats the locality Xīnpíng (Yunnan, China) and includes only the year of collection [19]80. This locality is separated from the closest one, Lùxī, by ca. 347 km (Fig 23).

Although some of the differences, notably those of the proportions of the ventrites and of the antennomeres, could be taken as enough to describe a different species, we prefer to wait until a series from the latter locality can be studied. These differences may be individual or populational, and the erection of a new taxon unjustified.

## Discussion

The genus Omophorus was placed in its original description between Tylomus Schoenherr, 1835 and Rhinaria W. Kirby, 1819, in Schoenherr’s Divisio Erirhinides, a conglomerate of unrelated long-nosed weevils with contiguous procoxae. The first genus is now a member of the tribe Sternechini Lacordaire, 1863 (Curculionidae: Molytinae) and the second is in Aterpini Lacordaire, 1863 (Curculionidae: Cyclominae) (Alonso-Zarazaga and Lyal 1999; Oberprieler 2010), two widely different and distantly related subfamilies. Lacordaire (1863) did not know the genus. In his description of its synonym Metatyges, Pascoe (1865) could not find a close relative, although he suggested a similarity to Haplonyx Schoenherr, 1836 (Curculionidae: Curculioninae, Cryptoplini) because of the short, stout form. Marshall (1917) placed his new genus Teluropus and Physarchus Pascoe, together with Omophorus, in a new subfamily Omophorinae, without mentioning the characters on which this taxonomic decision was based. This placement was also recorded in the World Catalogue (Schenkling and Marshall 1936). Kuschel (1987) placed Omophorini as a tribe of Molytinae, and synonymized Sternechosomini Voss, 1958 with it, without any further comment on the characters which substantiated this action, although this rank had already been used by Voss (1962).

Neither of the above mentioned authors made reference to H. Jekel (1873), who was the first to compare this genus with other taxa (which have remained similarly poorly known): Gonipterini Lacordaire, 1863, Rachiodes Schoenherr, 1835, Physarchus, and Sternechini Lacordaire, 1863, and with the more distant, in his opinion, Paipalesomus Schoenherr, 1847 and Peribleptus Schoenherr, 1843 (now considered to be synonyms), although based on characters which still need an evaluation.

There are no known detailed descriptions of the members of the genus Omophorus or of any other Metatygini. The most modern description is that of Omophorus boxi by Marshall (1944), who only gave a dorsal picture of the whole animal, the description being aimed at species recognition. We have benefited from the available quantity of material and completely dissected one male that lacked head and pronotum, to procure details on structures that, even if they are not useful for species recognition, may cast light on the phylogenetic relationships to other groups, a subject now in debate among specialists in the group.

Among the characters that can be of phylogenetic use, we want to emphasize the wing, the meso- and metanotal structures and the peculiar conformation of the female tergite VII.

The wing of Omophorus rongshu is very similar to that figured for Trigonocolus curvipes by Davis (2009, fig. 839), except for the strong loop of the union Cu-Cu1, the shortening of af, that does not meet the wing margin and is not bisinuate, the absence of 1A1 and 1A2, the longer rm and the more sclerotized 1rs and 2rs. They share the strong weakening of 3A. The apparent presence of 1A1 and 1A2 in Trigonocolus curvipes could be an artefact of Davis’s picture and could correspond in fact to thin folds, as they usually appear in wing preparations, since the wing surface is never perfectly flat. A strong loop in the union Cu-Cu1 is also visible in the wing of Cyllophorus fasciatus, a species probably not very close phylogenetically.

The meso- and metanota present similarities to some other taxa presented by Davis (*l.c.*). The broadly developed mesonotal axillary cord is a character commonly found in Baridinae, Trigonocolus and some Conoderinae, while a reduced axillary cord is found in some Curculioninae, Cossoninae, Ceutorhynchinae and some Conoderinae (the latter having been already considered probably paraphyletic in a phylogenetic analysis by Davis (2011)). The metanotum shows a peculiar widely rounded union of the posteromedial margin of metascutum and the allocrista, so that the anterior meeting point is distant from the front margin of the scutellar groove (in most species pictured by Davis the union forms an acute angle and the meeting point is precisely the point where allocrista and margins of scutellar groove meet anteriorly). This particular character is also found in Trigonocolus in a lesser degree. The strongly prominent, dentate axillary areas are, however, distinctive.

The dorsally uncovered VIII tergite of females (similar to that of male) is a distinctive character. It is exposed by the VII tergite being widely notched apically, an uncommon feature. So far, an uncovered female VIII tergite has been recorded only in Brachyceropseini, Ulomascini, Ectemnorhinini and Sitonini (Thompson 1992). The first tribe has been related to Lithinini in Molytinae (Oberprieler 2010), while the second is usually placed in Curculioninae. The third and fourth are in Entiminae, although the placement and rank of the latter have been questioned (Morimoto et al. 2006). The configuration of the terminal segments of abdomen in most of the hypothetically related taxa is unknown, and maybe this is an autapomorphy of the genus.

The distribution of some characters used in the systematics of Curculionidae is, so to speak, erratic and there is no certainty that their presence or absence is a proof of monophyly. Unci on the three pairs of tibiae are a common feature in several tribes in Curculioninae and they have been lost in some other groups (Cossoninae, Molytinae) in at least one pair (usually the hind one). Thus the presence of unci alone is a questionable synapomorphy to delimit monophyletic taxa (Curculioninae vs. Molytinae). The same can be said of the type 1 of stridulatory system (Lyal and King 1996) and of the sclerolepidia (Lyal et al. 2006). The hypothesis of the placement of the tribe Metatygini in Molytinae (Kuschel 1987) cannot be falsified with the data here presented. Some of the characters here presented point to possible relations to other tribes in and out of the Molytinae. McKenna et al. (2009) showed a phylogenetic tree based on Bayesian analysis in which the sister group of Scolytinae was a poorly resolved set of species belonging to several subfamilies of higher Curculionidae: Baridinae (*s.l.*), Curculioninae, Molytinae and Cossoninae. Even if the sampling was not complete, this result points out the complexity of a further analysis of the subdivisions. The two main branches of this higher group lacked support, the Molytini were found closely related to Curculio (no support) and to Cossonus (no support), while the Lixinae were placed near Baris. If this division into two groups deserves recognition, the Molytini Schoenherr, 1823 would have to be downgraded to a tribe of Curculioninae Latreille, 1802, and the Baridini Schoenherr, 1836 (*s.l.*) to a tribe of an extended Lixinae Schoenherr, 1823. However, no morphological characters are known supporting this division. On the other hand, a further effort should be made for the inclusion in the molecular phylogenies of species of the genus Rhamphus Clairville, 1798 and its relatives of the tribe Rhamphini Rafinesque, 1815, which has priority over the latter two family-group names, to achieve some nomenclatural stability when the molecular phylogenies had to be considered.

## Supplementary Material

XML Treatment for Omophorus

## Appendix I

Checklist of tribe Metatygini. The countries are in bold, the provinces in small capitals, general localities in inverted commas.

**Table:**

Genus Omophorus Schoenherr, 1835: 479 [type species: Omophorus stomachosus Boheman, 1835, by original designation]		
	Metatyges Pascoe, 1865: 424 [type species: Metatyges turritus Pascoe, 1865 by subsequent designation by Pascoe, 1870: 444; syn. Marshall, 1917: 195)	
(Omophorus)		
Omophorus (Omophorus) boxi Marshall 1944: 350		**Kenya:** Eastern: Kabete; Nairobi: Nairobi
Omophorus (Omophorus) cupreus Pascoe 1870: 443 (Metatyges)		**Benin; Ghana;** “widely distributed in West Africa” [Marshall 1944: 351]
	Omophorus (Omophorus) nicodi Hustache 1925: 22 (syn.: Marshall 1944: 351)	
Omophorus (Omophorus) hacquardi Chevrolat 1881: 89 (Metatyges) comb. n. (1)		**Tanzania:** Morogoro: Mhonda
Omophorus (Omophorus) indispositus Boheman 1845: 447		**Angola:** Benguela: Benguela; **D.R. Congo:** Bas-Congo: Boma, Banana-Boma; Sud-Ubangi: Zambi
	Omophorus (Omophorus) parvus Faust 1899: 416 (Metatyges) (syn.: Marshall,1917: 195)	
	Omophorus (Omophorus) occidentalis Fairmaire 1902: 135 (syn.: Marshall 1944: 351)	
Omophorus (Omophorus) stomachosus Boheman 1835: 480		**South Africa:** “Caffraria, Caffernland”
	Omophorus (Omophorus) turritus Pascoe 1865: 424 (Metatyges) (syn.: Marshall 1917: 195)	
(Pangomophorus) Voss 1960: 344 [type species: Omophorus biroi Voss, 1960, by original designation]		
Omophorus (Pangomophorus) biroi Voss 1960: 344		**Papua New Guinea:** Morobe: Sattelburg (= Sattelberg)
(Sinomophorus) Alonso-Zarazaga, Wang, Ren & Zhang, *h.o.*		
Omophorus (Sinomophorus) rongshu Alonso-Zarazaga, Wang, Ren & Zhang, *h.o.*		**China:** Yunnan: 潞西 (Lùxī), 瑞丽 (Ruìlì), 新平 (Xīnpíng) (?)
Genus Physarchus Pascoe 1865: 425 [type species: Physarchus pyramidalis Pascoe, 1865, by monotypy]		
Physarchus castaneipennis Faust 1891: 285		**India:** Sikkim
Physarchus conspicillatus Fairmaire 1878: 286		“Polynesia”
Physarchus pyramidalis Pascoe, 1865: 425		**Fiji**
Genus Sternechosomus Voss 1958: 46 [type species: Sternechosomus octotuberculatus Voss, 1958, by original designation]		
Sternechosomus octotuberculatus Voss 1958: 46		**China:** Fujian: Guàdūn 挂墩 (= Kuatun)

^1^ This species was overlooked by Schenkling and Marshall (1936). Moreover the species was named after the Père A. Hacquard, thus the original spelling *hocquardi* is incorrect and is corrected here under Art. 32.5.1 of the Code

## References

[B1] Alonso-Zarazaga MA Lyal CHC (1999) A World catalogue of families and genera of Curculionoidea (Insecta: Coleoptera) (Excepting Scolytidae and Platypodidae). Entomopraxis S.C.P., Barcelona, 315 pp.

[B2] Boheman CH (1835) In: Schenkling S (Ed) Coleopterorum Catalogus auspiciis et auxilio W. Junk, 150: Prionomerinae: 1–11; Aterpinae: 1–9; Amalactinae: 1–3; Haplonychinae: 1–8; Omophorinae: 1–2.

[B3] Boheman CH (1845) In: Schenkling S (Ed) Coleopterorum Catalogus auspiciis et auxilio W. Junk, 150: Prionomerinae: 1–11; Aterpinae: 1–9; Amalactinae: 1–3; Haplonychinae: 1–8; Omophorinae: 1–2.

[B4] Chevrolat A (1881) Description de Curculionides de Zanguebar. Annales de la Société entomologique de Belgique 25:85-94

[B5] Davis SR (2009) Morphology of Baridinae and related groups (Coleoptera, Curculionidae). ZooKeys 10:1-136

[B6] Davis SR (2011) Delimiting baridine weevil evolution (Coleoptera: Curculionidae: Baridinae). Zoological Journal of the Linnean Society 161 (1):88-156

[B7] Fairmaire L (1878) Diagnoses de Coléoptères des îles Viti, Samoa, Tonga, etc. Petites nouvelles entomologiques 2(210): 286

[B8] Fairmaire L (1902) Coléoptères nouveaux de San-Thomé et du Benguéla. Bulletin de la Société entomologique de France, 1902 (6):134-136

[B9] Faust J (1891) Curculioniden aus Ost-Indien. Entomologische Zeitung, Stettin 52 (7–12):259-287

[B10] Faust J (1899) Curculioniden aus dem Congo Gebiet in der Sammlung des Brüsseler königlichen Museums. Annales de la Société entomologique de Belgique 43:388-436

[B11] Hustache A (1925) Curculionides nouveaux de l’Afrique tropicale. Quatrième Partie. Annales de la Société linnéenne de Lyon (N.S.) 71:16-25

[B12] Jekel H (1873) Note sur les genres Peribleptus Sch., Paipalesomus Sch. et Paipalephorus Jekel. Annales de la Société entomologique de France, (5)2(4) [1872]: 433–442

[B13] Jha LK Sen-Sarma PK (2008) Forest Entomology. APH Publishing, New Delhi, 387 pp.

[B14] Kuschel G. (1987) The subfamily Molytinae (Coleoptera: Curculionidae): General notes and descriptions of new taxa from New Zealand and Chile. New Zealand Entomologist 9:11-29

[B15] Lacordaire T (1863) Histoire Naturelle des Insectes. Genera des Coleoptères ou exposé méthodique et critique de tous les genres proposés jusqu’ici dans cet ordre d’insectes. Vol. 6. Roret, Paris, 637 pp.

[B16] Lyal CHC Douglas DA Hine SJ (2006) Morphology and systematic significance of sclerolepidia in the weevils (Coleoptera: Curculionoidea). Systematics and Biodiversity 4(2): 203–241

[B17] Lyal CHC King T (1996) Elytro-tergal stridulation in weevils (Insecta: Coleoptera: Curculionoidea). Journal of Natural History 30:703-773

[B18] Marshall GAK (1917) On new species of Indian Curculionidae.- Part III. Annals and Magazine of Natural History, (8) 19 (110):188-198

[B19] Marshall GAK (1944) New East African Curculionidae. J. E. Afr. Ug. nat. Hist. Soc. 17: 308–354

[B20] McKenna DD Sequeira AS Marvaldi AE Farrell BD (2009) Temporal lags and overlap in the diversification of weevils and flowering plants. Proceedings of the National Academy of Sciences 106 (17):7083-7088 10.1073/pnas.0810618106 PMC2678426 19365072

[B21] Mittermeier RA Myers N Goettsch Mittermeier C (2000) Hotspots: earth’s biologically richest and most endangered terrestrial ecoregions. CEMEX, Conservation International, Mexico City, 340 pp.

[B22] Morimoto K Kojima H Miyakawa S (2006) Curculionoidea: General introduction and Curculionidae: Entiminae (Part1). Phyllobiini, Polydrusini and Cyphicerini (Coleoptera). The Insects of Japan. Vol. 3, 406 pp.

[B23] Oberprieler RG (2010) A reclassification of the weevil subfamily Cyclominae (Coleoptera: Curculionidae). Zootaxa 2515:1-35

[B24] Oberprieler RG Marvaldi AE Anderson RS (2007) Weevils, weevils, weevils everywhere. Zootaxa 1668:491-520

[B25] Pascoe FP (1865) On some new genera of Curculionidae. Part I. Journal of Entomology 2(13): 413–432, pl. XVII.

[B26] Pascoe FP (1870) Contributions towards a knowledge of the Curculionidae. Part I. Journal of the Linnean Society of London 10(47): 434–458 + pl. XVII.

[B27] Pascoe FP (1888) Descriptions of some new genera and species of Curculionidae, mostly Asiatic. Part V. Annals and Magazine of Natural History (6) 2 (11):409-418

[B28] Schenkling S Marshall GAK (1936) Curculionidae: Prionomerinae, Aterpinae, Amalactinae, Haplonychinae, Omophorinae. In: Schenkling S (Ed) Coleopterorum Catalogus auspiciis et auxilio W. Junk, 150: Prionomerinae: 1–11; Aterpinae: 1–9; Amalactinae: 1–3; Haplonychinae: 1–8; Omophorinae: 1–2.

[B29] Schoenherr CJ (1835) Genera et species curculionidum, cum synonymia hujus familiae. Species novae aut hactenus minus cognitae, descriptionibus a Dom. Leonardo Gyllenhal, C. H. Boheman, et entomologis aliis illustratae. Tomus tertius. Pars prima. [1836]. Parisiis: Roret; Lipsiae: Fleischer, 1–505 pp.

[B30] Taylor DE (1978) The fig weevil. Rhodesia Agricultural Journal 75(5): 119

[B31] Thompson RT (1992) Observations on the morphology and classification of weevils (Coleoptera, Curculionidae) with a key to major groups. Journal of Natural History 26:835-891

[B32] Velázquez de Castro AJ (1998) Morphology and taxonomy of the genus Sitona Germar 1817, (I): The metendosternite (Col., Curc.). In: Colonnelli E Louw S Osella G (Eds) Taxonomy, ecology, and distribution of Curculionoidea (Col.: Polyphaga). Proceedings of a Symposium (28 August, 1996, Florence, Italy). XX International Congress of Entomology. Atti del Museo Regionale di Scienze Naturali, Torino, 109–123

[B33] Voss E (1958) Ein Beitrag zur Kenntnis der Curculioniden im Grenzgebiet der orientalischen zur paläarktischen Region (Col. Curc.). Die von J. Klapperich und Tschung Sen in der Provinz Fukien gesammelten Rüsselkäfer. Decheniana, Beihefte 5:1-139

[B34] Voss E (1960) Die von Biró auf Neu Guinea aufgefundenen Rüsselkäfer, III (Coleoptera, Curculionidae). Annales historico-naturales Musei nationalis hungarici, Budapest 52:313-346

[B35] Voss E (1962) Attelabidae, Apionidae, Curculionidae (Coleoptera Rhynchophora). Exploration du Parc National de l’Upemba. Mission G. R de Witte 44:1-380

[B36] Zhou ZhK Gilbert MG (2003) Moraceae. Flora of China 5:21-73

